# Mobile Microfluidics

**DOI:** 10.3390/bioengineering6010005

**Published:** 2019-01-03

**Authors:** Mirela Alistar

**Affiliations:** Atlas Institute and Department of Computer Science, University of Colorado Boulder, Boulder, CO 80309-0320, USA; mirela.alistar@colorado.edu; Tel.: +49-151-7196-9187

**Keywords:** microfluidic biochips, mobility, healthcare

## Abstract

Microfluidics platforms can program small amounts of fluids to execute a bio-protocol, and thus, can automate the work of a technician and also integrate a large part of laboratory equipment. Although most microfluidic systems have considerably reduced the size of a laboratory, they are still benchtop units, of a size comparable to a desktop computer. In this paper, we argue that achieving true mobility in microfluidics would revolutionize the domain by making laboratory services accessible during traveling or even in daily situations, such as sport and outdoor activities. We review the existing efforts to achieve mobility in microfluidics, and we discuss the conditions mobile biochips need to satisfy. In particular, we show how we adapted an existing biochip for mobile use, and we present the results when using it during a train ride. Based on these results and our systematic discussion, we identify the challenges that need to be overcome at technical, usability and social levels. In analogy to the history of computing, we make some predictions on the future of mobile biochips. In our vision, mobile biochips will disrupt how people interact with a wide range of healthcare processes, including medical testing and synthesis of on-demand medicine.

## 1. The Premises of Microfluidics as Micro-Laboratories

**Figure 1:**
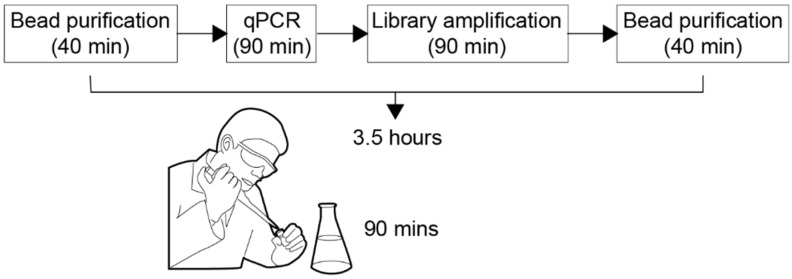
We depict schematically the bio-protocol called “Ovation Target Enrichment Technology for DNA”, developed by NuGEN Technology Inc. [1]. As illustrated, the duration of the bio-protocol is of 3.5 h, out of which 90 min of manual pipetting.

In the common practice of biological laboratories, the tasks composing the various stages of bio-protocols are separately performed by manual interfacing between them. Figure 1 illustrates, for example, the main stages of a typical bio-protocol. During a working day, lab researchers have to pipette fluids in tubes, carry them from one machine to another, individually program each machine, carefully document each step of the process and then convert the results into data before their analysis and validation. A major challenge in biology research is the reliable implementation of an automated and integrated workflow, which truly enforces bio-protocols reliability and reproducibility. An important step forward towards the solution of the problem is the integration of microfluidics into the laboratory workflow.

At the beginning of the 1990s, microfluidic technology progresses made possible the realization of the first examples of “micro total analysis systems”, demonstrating the possibility of automation, miniaturization and integration of complex biochemical protocols [2].

The trend today is toward *microfluidic platforms*, which according to reference [2], provide “a set of fluidic unit operations, which are designed for easy combination within a well-defined fabrication technology”, and offer a “generic and consistent way for miniaturization, integration, customization and parallelization of (bio-)chemical processes”. Microfluidic platforms are used in specific applications in healthcare such as drug discovery [3], diagnostic testing [4], prenatal screening [5], environmental monitoring [6], as well as optimizing laboratory procedures in molecular biology [7], enzymatic and proteomic analysis [8], single cell analysis [9,10], etc. Notable achievements are the microfluidic chips for HIV and syphilis testing [4], for non-invasive testing of chromosomal abnormalities [5], and for the discovery of the hepatitis C target and inhibitors [3].

Immediate advantages of microfluidics are *miniaturization*—minimizing the reagent consumption and time to result, *automation*—reducing the likelihood of human error, and *integration*—eliminating additional equipment for intermediate steps. 

Significant research efforts have been directed toward reducing the size of microfluidic platforms. Pressure-based microfluidics chips can integrate a million valves in an area less than the surface of a coin [11,12,13,14,15,16,17,18,19,20]. Digital microfluidic platforms can contain electrodes of 100 μm pitch that can transport 30 picoliter droplets [21]. Acoustic microfluidic chips are usually less than 1 cm in diameter and can manipulate droplets in the range of femtoliters [22]. The reduced size of microfluidic platforms contributes to their portability, making them ideal candidates for near-patient and point-of-care testing [23,24]. 

Moreover, due to miniaturization, the reagent and sample consumption is lower and the bioassay time-to-result is shortened. By using smaller volumes of expensive reagents and hard-to-obtain samples, the costs are significantly reduced and thus address an important concern for clinical laboratories. For example, acoustic microfluidic sensors cost as low as $1.50 for each disposable chip and can quantify the number of proteins bound to their surface [25]. These sensors have been successfully integrated into a microfluidic platform for HIV testing [26]. 

Faster reaction times are observed when using volumes at the microliter scale, making microfluidics suitable for flash chemistry applications [27,28]. Gray et al. [26] demonstrate that they can use acoustic microfluidics to test for HIV within 5 min (the usual time is 24 h). Sista et al. [29,30] developed a digital microfluidic platform to screen newborns for lysosomal storage diseases. Their work showed that the testing time for Pompe and Fabry disorders can be significantly reduced from 2 days to 120 min by using digital microfluidics. These great improvements are the direct result of using smaller amounts of fluids. At microfluidic scale, the diffusion distance is significantly smaller, thus reducing the time needed for the complete mixing of droplets, and consequently, triggering faster biochemical reaction times.

Last, microfluidic platforms are programmable, i.e., that they can be controlled automatically, by algorithms, and thus adapt real-time to various needs. The programmability aspect of microfluidics has been explored for high-throughput execution of repetitive bio-protocols such as sample preparation [31,32,33]. However, the history of computing taught us that there are more advantages of automation then high-throughput. In the future, we may be able to build large-scale systems based on microfluidics, with networking and communication capabilities, similar to today’s distributed computer systems. 

**Figure 2:**
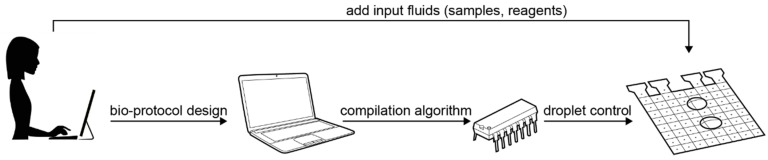
The automated setup for executing a bio-protocol on a digital microfluidic biochip: after the bio-protocol is designed, it is compiled automatically into an “electrode actuation sequence”, which controls the movement of droplets to run the bio-protocol. The droplet control instructions are stored on a microcontroller and triggered after the biochip has been loaded with the required fluids.

Even more, as schematically depicted in Figure 2, microfluidic platforms may become personal instruments, enabling everyone to design and test their own bio-protocols through the means of advanced automation algorithms. The types of personal bio-protocols can vary in their purpose and complexity, from fundamental chemical experiments for educative purposes, to complex bio-protocols serving a medical need. We envision people using microfluidic platforms to extract pigments, mix perfumes, and cook molecular drinks, but also to design their optimal hormonal contraceptive and even to mix a phage cocktail targeting their bacterial infection. 

## 2. The Future: Mobile Microfluidics

Microfluidic platforms implement split, merge, and mix operations to manipulate fluids containing chemical compounds, and such a process is somehow analogous to how a computer chip combines low level mathematical operations such as additions, multiplications and logical operations to numbers and data, in order to execute programs. Since microfluidic platforms implement the execution of a series of wet-lab tasks, they have thus the potential to represent and run bio-protocols as if they were “computer programs”. Thus, if bio-protocols can be fully described as programs and microfluidic platforms can act as “execution units”, then a bio-protocol execution can be fully formalized and automatized like any software execution.

Given that we placed the future of microfluidics, and by extension healthcare, into a digital sphere, it is worth taking a look at the history of computing. In terms of their roadmap, computers evolved from the room-size Electronic Numerical Integrator and Computer (ENIAC) [34], to desktop computers, laptops, tablets and smartphones. The development in computing technology is currently focused on *mobile*, with significant efforts invested in embedding computation in wearables [35]. 

Similar to how mobile computing has enabled over 60% of the population to solve a wide range of problems (e.g., navigation, information retrieval) by means of software, we argue that microfluidic platforms can change how people interact with a wide range of healthcare processes, including medical diagnosis and synthesis of on-demand medicine. Indeed, significant research is being conducted to develop microfluidic platforms that are operable even in remote areas. Ng et al. [36] developed a microfluidic platform that tested children and adults for rubella and measles in a refugee camp in Kenya. Several research groups work on malaria detection using microfluidic platforms [37,38,39,40].

**Figure 3:**
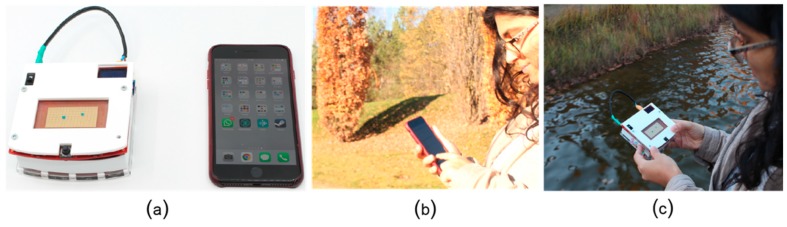
Transitioning towards mobile microfluidics implies that the instruments are operated from a battery (**a**), hand-held (**c**) and used in similar contexts as a smartphone (**b**,**c**).

In this paper, we investigate how far the development of mobile microfluidics can be pushed, and specifically, what needs to be done so that they impact our lives the same way mobile computing did. So far, all microfluidic platforms tested in remote areas are point-of-care systems, i.e., they are used in a controlled setting (e.g., someone’s house, a tent, an ad-hoc clinic) and operated by experts. While microfluidic platforms hold the promise of unlocking healthcare for people that do not have access to it, such as the Kenyan refugees, we wonder how the *first-world society* will be impacted if microfluidic platforms achieve mobility. Specifically, if microfluidic platforms transition from point-of-care systems to mobile. Similar to smartphones, microfluidic platforms have to be more “compact and portable”, they have to be hand-held devices, more than “field-deployable”, they have to be operated off-grid (e.g., from a battery), and also in the absence of experts in unexpected situations such as during sport activities or camping trips (Figure 3). Real-time response, such as automatic fault handling based on sensor output, is also crucial to ensure the microfluidic platform adapts to the unpredictability of mobile use. 

We argue that mobile microfluidics are far more reaching than providing access to medical procedures. If mobile microfluidics succeeds as mobile computing did, then microfluidic platforms will have a similar seductive power to make their use personal and intimate. The revolutionary change we envision lays in the fact that people will start *practicing* healthcare through daily habits, thus replacing the last-minute doctor visits. Mobile microfluidics strongly connects computing and health: the platform can perform bio-protocols to monitor our physiological fluids, as well as help us check progress through notifications and feedback. 

## 3. Current Trends in Microfluidics Research

Microfluidic platforms can be classified according to the liquid propulsion principle used for operation, e.g., capillary, pressure driven, acoustic or electrokinetic. In Table 1, we present an overview of the four types of microfluidics, comparing them in terms of their size, programmability, mobility and at-home use. As shown in column 5, the only two categories that have been explored for mobility are capillary and electrokinetic microfluidics. Since the capillary platforms are not programmable, we focus on electrical biochips in subsection “*3.3 Candidates for Mobile Microfluidics”* and present our findings after testing the microfluidic platform with the smallest size biochip, OpenDrop [41], in various mobile scenarios. 

### 3.1. Fluid Actuation on Microfluidic Platforms

This section offers an overview of the existing microfluidic platforms. In the following paragraphs, we discuss each one of the four categories by identifying the most known examples and assessing their usage in the mobile context. 

**Figure 4:**
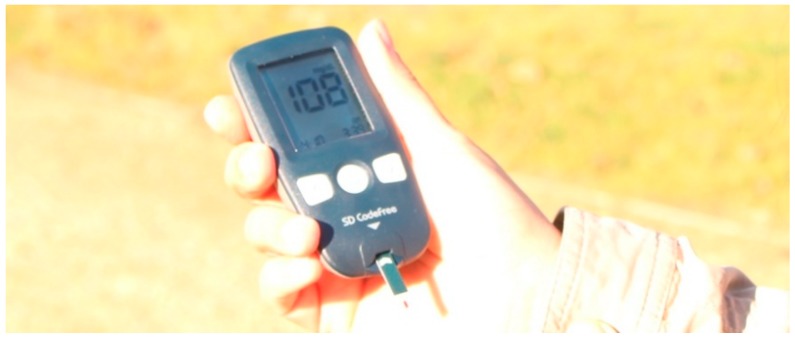
A glucose meter is a commercial instrument that uses capillary microfluidics to measure glucose levels in blood droplets. It is operated from a battery and can be used in mobile scenarios.

The capillary force can manipulate fluids solely based on the surface tension between them and the solid substrate. The main use of capillary forces is in paper microfluidics. Usually, such microfluidic platforms consist of paper strips previously primed with reagents and covered in plastic to avoid contamination. The most common used capillary platform is the pregnancy test, which uses a strip that, after dipped in urine, indicates through color whether the user is pregnant or not. These tests are robust, cheap and can be used at home. Other common applications of paper microfluidics include the widely spread tests for vaginal infections such as chlamydia [45] and glucose measurements in blood samples [46] (Figure 4). Recently, research started to explore the potential of capillary microfluidics as wearables, or so called “labs-on-skin” [47,48]. Examples include smart wound bandages [49] and a soft stretchable arm patch that measures compounds found in sweat [50]. While these tests provide cheap ad-hoc diagnosis, they fail to explore the programmability aspect of microfluidics. To overcome that, recent developments combine paper microfluidics with the computational power of smartphones, e.g., using their cameras to read the results for HIV tests [51,52] and pathogen tests in urine [53,54].

**Figure 5:**
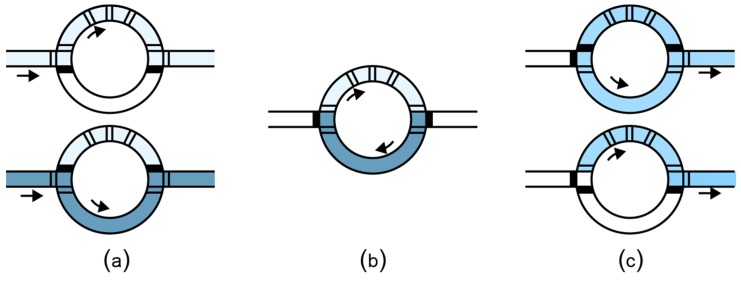
Mixing in a flow channel microfluidic chip. (**a**) The lower valves are closed allowing the first fluid to fill the upper part of the mixer. Similarly, by closing the upper valves and opening the lower valves, the second fluid enters the chip. (**b**) The valves inside the rotary mixer are actuated one by one, thus generating a flow that mixes the fluids. (**c**) The mixed fluid exits the rotary mixer.

Pressure-driven microfluidics manipulates fluids in micro-channels. On such platforms, miniature pressure valves control the flow of fluids in the channels, allowing two different fluids to be transported and mixed as shown in Figure 5. 

**Figure 6:**
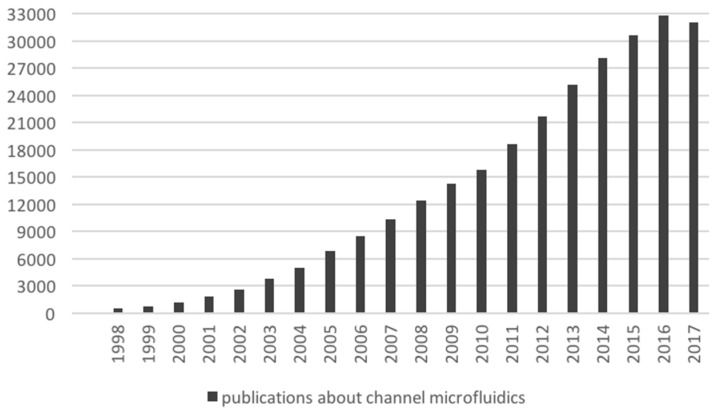
Publication count related to channel-based microfluidics. The count was retrieved from Google Scholar, by differential search between the topics “microfluidic” and “electrowetting microfluidics”. The values reported in this figure exclude patents and citations.

Pressure-driven platforms currently attract the most research efforts in the microfluidics community, with an average of 30,000 papers per year since 2015 (see Figure 6 for an overview of the publication trend in channel-based microfluidics). Comprehensive surveys cover the existing microfluidics research in cell analysis [55,56,57], Caenorhabditis elegans modeling [58,59,60], organ-on-chip [61,62,63,64], infectious diseases [65] and point of care diagnosis [66,67,68]. 

Unfortunately, in spite of the impressive reduction in the size of the valves (approximately 1 million valves can be packed in 1 cm^2^ [11]), flow-channel microfluidics are not suitable for mobile use. The miniature chips require a complex setup of tubes and pumps, and it was observed that they became “chips-in-a-lab”, rather than the awaited “labs-on-chip” [69]. 

**Figure 7:**
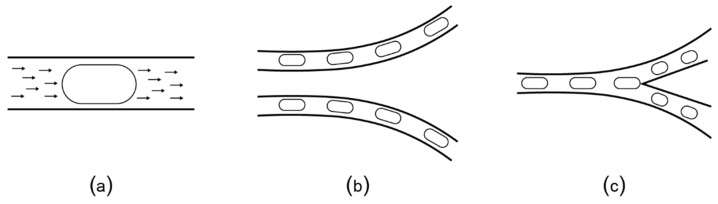
(**a**) Plugs (elongated droplets) are transported by the flow of the carrier oil that is continuously pumped through the micro-channels. (**b**) Plugs follow the direction of the oil flow along the channel, unless (**c**) there is a bifurcation that causes the plug to split.

Two-phase flow microfluidic platforms use a similar setup as the valve-controlled channels to manipulate individual droplets (also called “plugs”) by dispensing the target fluid into a carrier fluid, such as oil. The fluid plugs are transported by pumped oil (Figure 7a) without the need of the micro-valves to control the flow. Depending on the geometry of the channels, the plugs can turn right or left (Figure 7b) or split (Figure 7c). The experimental setup is easy to build and can be powered by a regular socket plug (i.e., not a pressure plug). Because of their usability, these chips are preferred for a series of applications such as single-cell assays [70,71], magnetic particle washing of bacteria from blood [72,73], and of fungi from blood [74]. Researchers from computer science developed algorithms to automatically design channel layouts optimized for specific applications [75,76].

**Figure 8:**
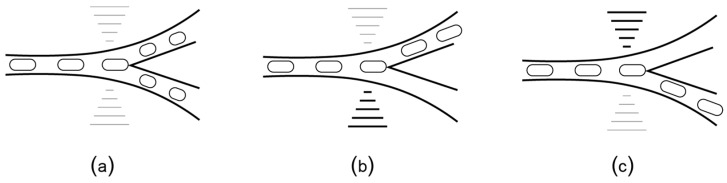
(**a**) SAW biochips add interdigitated transducers orthogonal to the flow channels. When positioned at a bifurcation, the transducers can generate acoustic waves (similar to micro-earthquakes) that direct the plugs towards (**b**) the left channel or (**c**) the right channel.

Recently, Stephenson [42] used small-size solenoid pumps to develop “MiniDrops”, a portable two-phase flow microfluidic instrument. MiniDrops has an integrated microscope and it is currently used at New York Genome Center Innovation Lab for single-cell RNA sequencing of rheumatoid arthritis synovial tissue. With further engineering, MiniDrops can be significantly reduced in size and adapted to run from a battery, making it suitable for mobile scenarios. Even so, two-phase flow microfluidic platforms are limited in terms of their adaptability: they are passive, i.e., the pathway of the plug is determined by the layout of the channels and cannot be changed at runtime. Microfluidic platforms can also use acoustic forces to manipulate fluids. Similar to the channel microfluidics, the droplets are used as “plugs”, in a two-phase flow setup. What is different is a set of additional interdigitated transducers positioned orthogonally to the micro-channels (Figure 8a). The transducers can be programmed to emit surface acoustic waves (SAW) at high frequency and low amplitude. These waves act like micro-earthquakes and can direct the droplet towards a specific channel, as shown schematically in Figure 8b,c. The research in SAW microfluidics is still in its incipient phase, with most of the work focused on fabrication [77,78] and theoretical modeling [79,80]. Notable results are the prototypes for HIV testing [26] and Pseudomonas aeruginosa detection [81]. According to the most recent survey, the trend is towards diagnosis and treatment of infectious diseases [82,83]. 

SAW-based chips have not been shown yet in a portable setting. We can envision a mobile platform based on SAW chips that uses solenoid pumps (similar to MiniDrops [42]) to inject oil and a surface-mount electronic circuit to generate and control the waves. Moreover, SAW-based chips are active and can make better use of the channel geometry, allowing them to adapt better to mobile use. 

**Figure 9:**
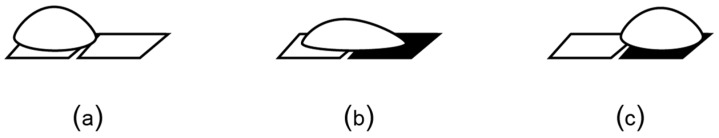
An EWoD biochip transports droplets on an array of electrodes. (**a**) In the absence of voltage, the droplet does not wet the surface due to the hydrophobic layer that coats the electrode. (**b**) Electrical voltage unbalances the force equilibrium at the solid-liquid-vapor interface, causing the droplet to wet the surface. (**c**) Consequently, the droplet moves toward the charged electrode.

Microfluidic platforms can also manipulate the liquids as droplets, using electrokinetics. As shown in Figure 9, droplets can be moved on an array of electrodes by applying an electrical voltage to the target electrode, phenomenon known as “electrowetting-on-dielectric” (EWoD) [84]. These microfluidic platforms use fluids solely as individual droplets, and not as flow, thus earning the name of “digital” biochips [85,86]. The electrodes can be programmed to bring the droplets to the same location and then mix them by moving them together in a specific pattern. The droplets larger than two electrodes can be split by actuating electrodes on both sides of the droplet (to pull the droplet in two opposite directions). 

Digital biochips are the most suitable for mobile use because they are small, programmable and can be operated by at-home users [41]. The remainder of the paper focuses on digital microfluidics, presenting its evolution, evaluating the current state of the art and proposing a realistic roadmap toward achieving mobility.

**Figure 10:**
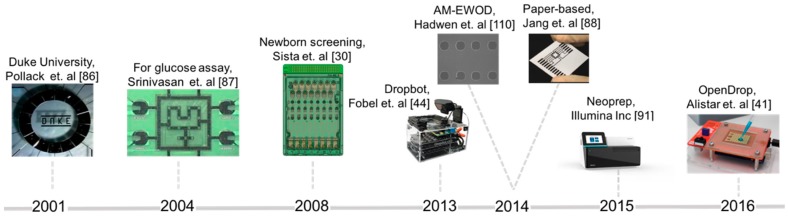
Illustration of the evolution of digital microfluidic biochips in the last years (from left to right): test microfluidic mixer developed by Duke University, a biochip for glucose assay, a device for parallel testing of 8 newborns for Pompe and Fabry diseases, DropBot—a generic do-it-yourself platform using chromium based electrodes, AM-EWOD biochip containing large array of electrodes (64 × 64) fabricated using thin-film transistor technology, biochips printed on paper using carbon nanotube ink, Neoprep—a benchtop device using digital microfluidics for sample preparation, OpenDrop—a cheap do-it-yourself biochip using printed circuit board technology.

Over the years, digital microfluidic platforms evolved from simple mixers to generic platforms that enable the execution of stages composing complex bio-protocols. As schematically depicted in Figure 10, Duke University developed in 2001 the first digital microfluidic biochip [86] capable of mixing droplets in a circle module made of electrodes. The prototype was further developed to integrate automatic dispensers and optical detectors [87]. Another breakthrough was achieved in 2010, with the introduction of biochips as benchtop devices with disposable cartridges [29]. This prototype was further developed by Advanced Liquid Logic [30] for fast and effective newborn screening in clinics and hospitals. Wheeler’s lab from Toronto University [44] and Shin’s lab from Sogang University [88] proposed in 2014 the use of printable paper-based electrodes for biochips, lowering significantly the cost per cartridge. In 2016, OpenDrop [41] was designed as a “do-it-yourself” device and released in the context of the open-science movement DIYBio. 

Specifically, relevant results, validated by multiple research groups, were obtained for three bio-protocols: the glucose assay [86,89,90,91,92], showing the potential for point-of-care testing, the Pompe and Fabry screening [30], showing the potential for diagnosis, and sample preparation [93], showing the potential to increase the throughput of laboratory workflows and procedures. In June 2017, Madison et al. [94] showed that biochips can be used to modify genetically E. coli, thus opening a new application area for digital microfluidics called “synthetic biology”. All such bio-protocols have been tested in the context of nanotechnology laboratories, generally exploiting the miniaturization advantage provided by microfluidics technology. 

### 3.2. From Fluid Actuation to Bio-Protocol

Microfluidic platforms have also the potential to benefit from design automation technology, an observation that stirred the interest of the computer engineering community. The trend so far was to adapt design automation techniques that were well-established in other fields such as large-scale integration of integrated circuits, for employing them in microfluidic systems. Specifically, recent works have introduced bio-protocol compilers [95,96,97,98], error correction algorithms [99,100] and the concept of synthesis of the physical device [93,101,102]. 

**Figure 11:**
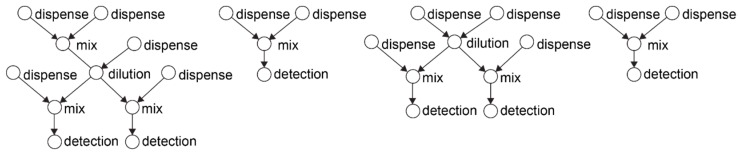
Example in-vitro diagnosis bio-protocol modeled as a direct acyclic graph. In-vitro diagnosis is a template bio-protocol for identifying the microbes in the human samples using genetic testing. This bio-protocol performs a series of dilutions with specific reagents that trigger a colorimetric reaction. The microbial concentration is optically detected, by measuring the absorbance of the reaction product [103].

All automation algorithms proposed so far are based on a graph model [99,100] that captures the bio-protocol as a sequence of fluidic operations represented by the nodes in the graph and their dependencies, the edges in the graph. Figure 11 illustrates an example in-vitro bio-protocol graph.

**Figure 12:**
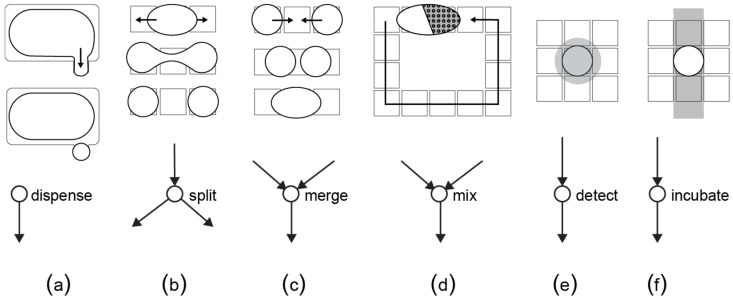
Each fluidic operation is modeled as a node with inputs and outputs. (**a**) A dispensing operation is a node with no predecessors, (**b**) a split operation divides one droplet into two equal daughter-droplets, (**c**) a merge operation combines together two droplets, (**d**) a mix operation transports a merged droplet over a specific route in order to achieve homogenous concentration, (**e**) a detection operation reads out a certain property of the droplet by means of an external sensor and (**f**) an incubation operation keeps the droplet at a constant temperature. For the detection and incubation, the biochip needs additional sensors and a temperature bar, respectively.

The graph is given as input to a compiler algorithm that transforms the graph operations into fluid movements. Concretely, the compiler calculates the best configuration of the biochip for each of the fluidic operations, and eventually outputs the control sequence needed to complete the bio-protocol [93]. This step depends on the type and architecture of the microfluidic platform. For example, a digital biochip can perform the basic fluidic operations as shown in Figure 12. The actuation sequence is stored in a microcontroller and triggered when the biochip is ready to run a bio-protocol, i.e., after the target fluids have been dispensed in the cartridge. 

In the context of mobile microfluidics, such algorithms can be used to adapt to unpredictable scenarios, such as system failure or misuse. Transient faults (i.e., the droplets are stuck) can be detected using sensor measurements and then automatically corrected by re-compiling the bio-protocol graph to include the recovery operations [104]. For example, once detected, a faulty split can be corrected by stopping the execution bio-protocol, re-mixing and re-splitting the droplets. A more complex failure may need the complete re-creation of the faulty droplet.

### 3.3. Candidates for Mobile Biochips

To qualify for mobile use, a microfluidic platform has to be small in size (ideally hand-held), programmable, operable off-grid, and easy to use at home. Apart from satisfying this minimum set of requirements, mobile microfluidic platforms have to overcome a series of technical and usability challenges. Moreover, to achieve revolutionary change, mobile microfluidic platforms need to be adopted at a large scale by the consumers, a milestone that involves solving specific ethical and social challenges. In this subsection, we present the results of a sanity screening of existing platforms and identify the ideal candidates for mobile microfluidics. In the next section, “The Roadmap to Mobile Microfluidics”, we derive the technical, usability and society challenges, based on the history of the mobile phone as well as our own experience with mobile biochips. 

As seen in Table 1, pressure-based microfluidic platforms have yet to be developed in sizes that can be hand-held and capillary microfluidics are not programmable. SAW biochips are extremely small, and based on their operation requirements, with some engineering efforts can be powered from a battery. In spite of being great candidates for mobile use, the research related to SAW biochips is still in its incipient phase and so far, there has not been any work to demonstrate their off-grid use.

**Figure 13:**
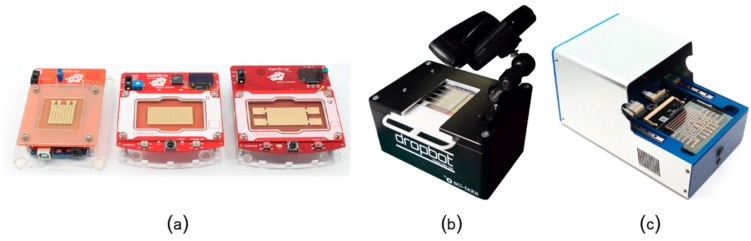
Example digital microfluidic platforms: (**a**) the three versions of OpenDrop [41] developed by Gaudilabs [105], (**b**) the latest version of DropBot [44] developed by Sci-Bots Inc. [106], and (**c**) the instrument developed by DigiBio B.V. [43]. The Dropbot photo is from R. Fobel and used with permission.

As the comparison in Table 1 shows, digital microfluidic platforms are currently the most suitable platforms for mobile scenarios and also the only programmable microfluidic platforms that have been tested for at-home use. Alistar [41] reports 72 users that tried to replicate and use OpenDrop instrument (Figure 13a) at home during the years of 2015-2017. The user range varied from engineers to artists and designers, with diverse interest also in using the platform for perfume mixing, information display, diagnosis and DNA computing. DigiBio instrument (Figure 13c**)** and DropBot (Figure 13c) are also within a portable size range, however, both platforms target scientists and researchers and have, thus, not been tested for at-home use. 

**Figure 14:**
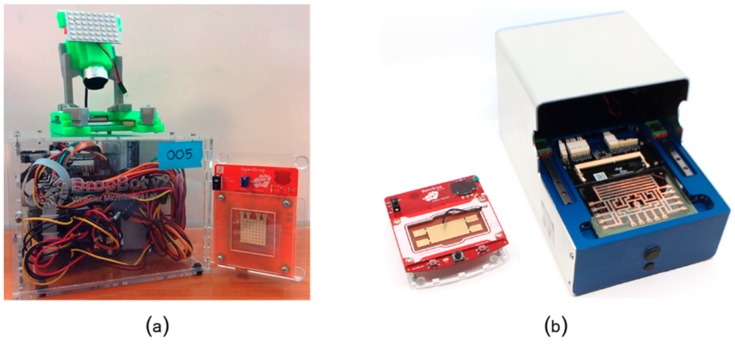
Direct comparison in size between (**a**) DropBot version 1 and OpenDrop version 1, and (**b**) OpenDrop version 3 and DigiBio instrument. The Dropbot photo is from R. Fobel and used with permission.

In Figure 14, we show a direct comparison between OpenDrop version 1 and DropBot version 1 (Figure 14a), and between OpenDrop version 3 and the DigiBio instrument (Figure 14b). Because of the relatively small size (these biochips can be easily held in hand), we chose to adapt OpenDrop for mobile use and give it a sanity test. The next section presents the lessons learnt from our experiment. 

## 4. The Roadmap to Mobile Microfluidics

### 4.1. Technical Challenges

**Figure 15:**
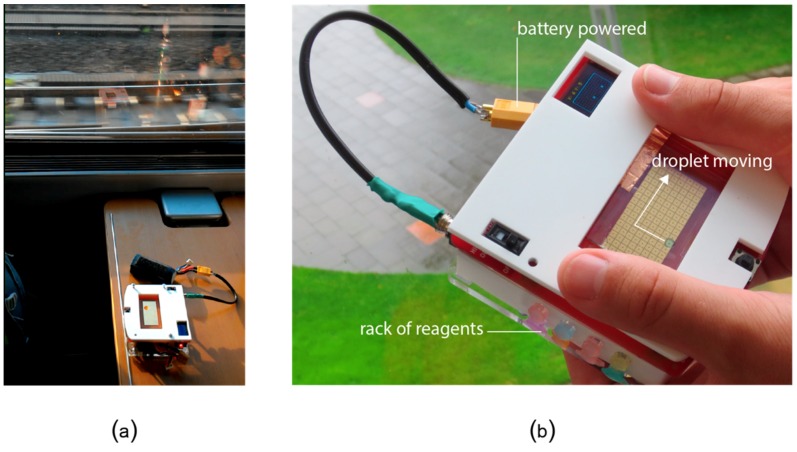
(**a**) Evaluating the robustness of OpenDrop under continuous shaking during a train trip. (**b**) Testing the water quality of a creek on a camping trip. We equipped OpenDrop with batteries, a cover to prevent damage to the electrodes and a cartridge with different reagents for basic health tests while on the go.

To explore the vision of mobile microfluidics, we adapted OpenDrop version 2 [41] for mobile use, i.e., we added a battery, casing, and reservoirs for reagents (Figure 15b). We then used the resulting instrument to execute a bio-protocol during traveling on a train ride (Figure 15a). We chose a water test quality from Aquanatura [107], which uses the chromogenic substrate 5-bromo-4-chloro-3-indolyl-beta-D-galactopyranoside (X-GAL), to detect the presence of coliform bacteria. We adapted the bio-protocol for microfluidic volumes. As a side effect of reducing the volumes from 100 mL to 2.5μl, the bio-protocol now ran in 1 h (instead of the original 48 h). 

#### 4.1.1. First Challenge: Robustness under Shaking and Tilting

During mobile use, microfluidic platforms can endure sudden (intense) shakes and extreme tilting, causing the droplets to slip off their trajectory. To overcome this, the platforms need to operate under a feedback control system that allows them to adapt in real-time. In our study case, OpenDrop should be equipped with an accelerometer that permanently records the position of the chip. The control software would use these measurements to respond to changes ad-hoc, e.g., by increasing the switching delay to allow the droplets enough time to reach the gap between electrodes and stabilize.

Our quick experiments with various switching delays showed their direct impact on droplet movement. For our specific setup, we used 400 ms for the smooth parts of the train ride and increased to 800 ms for the most intense shakes. A downside is an operation speed reduction of 50%. 

OpenDrop can be further equipped to sense the exact position of the droplets and adapt to eventual slips by re-positioning the droplets on their initial trajectory. We briefly explored this idea by reprogramming a parallel redundant path to capture the slipping droplets. 

**Figure 16:**
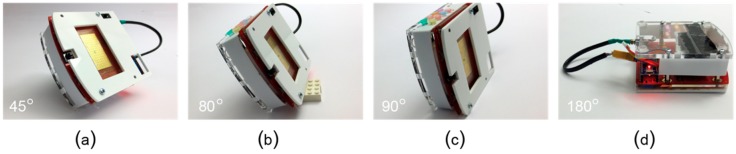
The experimental conditions (**a**–**d**) tilting the device in different orientations, at 45, 80, 90 and 180 degrees respectively.

OpenDrop, similar to previous work, such as Droplet-on-a-Wristband [108], can manipulate water droplets against gravity. In the case of our sanity check, the challenge was to manipulate droplets with much larger density and lower surface tension than deionized water. During our tests, we found that the open (i.e., uncovered) biochip can successfully work under angles of maximum 10 degrees. At larger angles it would fail, as the droplets are too heavy to be pulled by the electrodes. We reduced the size of droplets from 23 μL to 2.5 μL by adding a top electrode at a gap height of 205 μm. As shown in Figure 16, the covered biochip can take angles of 80 degrees. When positioned at 90 degrees sideways, the droplets started to slip slowly and eventually went off the trajectory. The covered biochip worked successfully when kept upside down, while the droplets fell off the single-sided biochip. 

#### 4.1.2. Second Challenge: Reducing Costs

Currently, OpenDrop costs $150 to produce, on top of that there are costs per reaction of $0.1 (for reagents). Occasionally (after 5–8 reactions) the top electrode needs replacement, which would add about $2 to the cost per reaction. The cost of the device in particular could substantially benefit from mass-production and further engineering. The reaction costs would remain comparable.

#### 4.1.3. Third Challenge: Reducing the Size

Currently the device measures 100 × 100 × 33 mm^3^, which is the size of the OpenDrop instrument. The size can be reduced by removing excess empty volume and minimizing some of the circuitry. This would compress the entire device down to about 100 × 70 × 20 mm (given the current electrode array). At current scale the device fits in a purse/bag, this could be further reduced to pocket size (e.g., comparable to a phone). This could be pushed even further by running the controls on phone hardware and varying the size of the electrode array.

#### 4.1.4. Fourth Challenge: Safety

Most digital microfluidic devices operate at an electrical voltage between 90 V to 300 V DC. In the context of outdoor use, securing the electronic components becomes imperative. Alternatively, mobile biochips can be fabricated using other technologies such as thin film transistor (TFT)-based electrodes and complementary metal–oxide–semiconductor (CMOS) technology [109,110], that recently demonstrated moving droplets at only 20 V DC. 

Another safety challenge concerns the use of bio-materials. Here, mobile microfluidics can get inspiration from its most successful applications: glucose meters and capillary tests. Sealed tubes with reagents and sterile pricking needles for collecting the samples are some of the options that have been implemented in the capillary tests and glucose meters. 

### 4.2. Usability Challenges

In terms of usability, the main challenge is embedding the domain knowledge into the microfluidic platform. In extreme situations, a mobile microfluidic platform will have to be able to guide the user to make the right interpretation of the bio-protocol result without the experience of a doctor. Currently, researchers tend to look at part of the solution, specifically at technical aid through smart sensors and automation. The overlooked aspect is *training* users, prior to mobile use, through repetitive exercises while on “safe” non-mobile settings, e.g., at home, or in the doctor’s cabinet.

Other notable challenges at the usability level are integrating networking capabilities into biochips and developing an appropriate communication protocol that ensures fault-free operation and secure data coordination. In this context, security means ensuring the correctness of the medical results, data authenticity, preventing attacks and avoiding misuse.

### 4.3. Society Challenges

Technological progress happens when people adopt at large the new technology and, historically, that depended on how ready the society is to trust the new technology. Transitioning to mobile microfluidics as a replacement for doctors’ cabinets may be too abrupt to be accepted by society. It has to be built on the recently introduced “e-doctors”, i.e., medical experts that consult their patients remotely, over the internet. Such e-doctors can be constantly in touch with the users of mobile microfluidic platforms and intervene when their progress is not satisfying. 

Regardless of how much people will trust mobile microfluidics, the questions of responsibility and liability in case of misuse remain open and will be solved in conformance with the ethical and legal stage of the society at that specific moment. 

## 5. Discussion and Conclusions

We presented the vision of using mobile microfluidic biochips as a means to provide healthcare to the broad population, even in situations that cannot be anticipated, such as during sports or outdoor activities. They could also be used far away from a doctor, in rural areas. 

**Figure 17:**
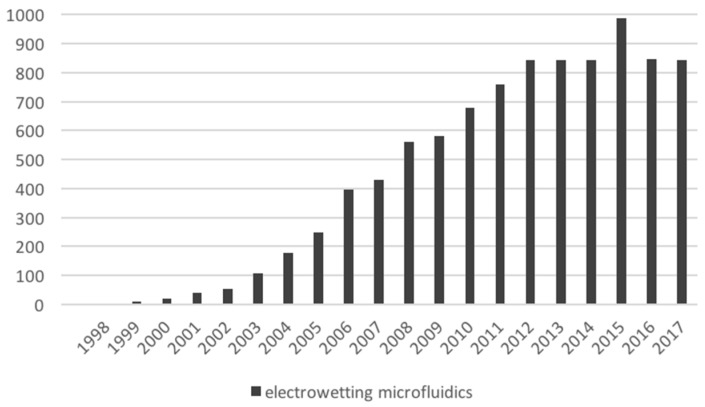
Publication count related to digital microfluidics. The count was retrieved from Google Scholar, by searching for “electrowetting microfluidics”. The values reported in this figure exclude patents and citations.

Out of the four major types of microfluidic platforms, digital biochips have the greatest potential to enable medical testing in situ—a first step toward enabling more effective preventive healthcare. We derived, based on our tests using OpenDrop during a train ride, the main technical challenges that need to be overcome by microfluidic research in order to achieve true mobility in healthcare. Unfortunately, in the last years, research in digital microfluidics seems to have dwelled in a comfortable zone, a fact shown by the stagnating number of publications (Figure 17). 

We argue that mobile use of microfluidics is not only a matter of efficiency, and it can expand beyond a tool to deliver drugs faster and cheaper. Mobile microfluidics can revolutionize the way we perceive healthcare by transforming it into a sum of our daily routines. To achieve that kind of intimate interaction with healthcare, microfluidic platforms need to get deeply embedded in our lifestyle in a similar way that mobile phones have achieved. 

In conclusion, we encourage microfluidic researchers to step out of the comfort zone and build bridges with complementary disciplines, in order to address the usability and society challenges needed to achieve true mobility.

## Figures and Tables

**Table 1:** Overview of the four types of microfluidic platforms.

Fluid Propulsion	Example	Size	Programmable	Off-Grid	At-Home
Capillary	Pregnancy test	Small	No	Yes	Yes
		(10 × 2 cm)			(>10^6^ users)
Pressure	MiniDrops [42]	Medium	Yes	Not	Not
		(15 × 15 × 10 cm^3^)		explored	Tested
	Quake-valve chips [11]	Very small	Yes	No	Not
		(5 × 3 × 1 cm^3^)			Tested
Acoustic	SAW biochips [26]	Very small	Yes	Not	Not
		(2.5 cm)		explored	Tested
Electrical	DigiBio Unit [43]	Large	Yes	No	Yes
		(15 × 25 × 15 cm^3^)			(<10 users)
	DropBot [44]	Medium	Yes	Not	Yes
		(15 × 20 × 10 cm^3^)		explored	(<20 users)
	OpenDrop [41]	Small	Yes	Yes, we tested	Yes
		(10 × 15 × 3 cm^3^)			(>70 users)
